# Antibacterial Properties and Computational Insights of Potent Novel Linezolid-Based Oxazolidinones

**DOI:** 10.3390/ph16040516

**Published:** 2023-03-30

**Authors:** M. Shaheer Malik, Shaikh Faazil, Meshari A. Alsharif, Qazi Mohammad Sajid Jamal, Jabir H. Al-Fahemi, Amrita Banerjee, Arpita Chattopadhyay, Samir Kumar Pal, Ahmed Kamal, Saleh A. Ahmed

**Affiliations:** 1 Department of Chemistry, Faculty of Applied Sciences, Umm Al-Qura University, Makkah 21955, Saudi Arabia; maasharif@uqu.edu.sa (M.A.A.);; 2 Department of Chemistry, Poona College of Arts, Science and Commerce, Pune 411001, India; 3 Department of Medicinal Chemistry and Pharmacology, CSIR—Indian Institute of Chemical Technology, Hyderabad 500007, India; 4 Department of Health Informatics, College of Public Health and Health Informatics, Qassim University, Al Bukayriyah 52741, Saudi Arabia; m.quazi@qu.edu.sa; 5 Department of Physics, Jadavpur University, 188, Raja S.C. Mallick Rd., Kolkata 700032, India; amritabanerjee30@gmail.com; 6 Technical Research Centre, S. N. Bose National Centre for Basic Sciences, Block JD, Sector III, Salt Lake, Kolkata 700106, India; 7 Department of Basic Science and Humanities, Techno International New Town, Block—DG 1/1, Action Area 1, New Town, Rajarhat, Kolkata 700156, India; arpita.chattopadhyay@gmail.com; 8 Department of Chemical and Biological Sciences, S. N. Bose National Centre for Basic Sciences, Block JD, Sector 3, Salt Lake, Kolkata 700106, India; skpal@bose.res.in; 9 Department of Pharmacy, Birla Institute of Technology and Science-Pilani, Hyderabad 500078, India; 10 Chemistry Department, Faculty of Science, Assiut University, Assiut 71516, Egypt

**Keywords:** oxazolidinone, linezolid, antibacterial, molecular modeling, molecular dynamics, ADME properties

## Abstract

The mounting evidence of bacterial resistance against commonly prescribed antibiotics warrants the development of new antibacterial drugs on an urgent basis. Linezolid, an oxazolidinone antibiotic, is a lead molecule in designing new oxazolidinones as antibacterial agents. In this study, we report the antibacterial potential of the novel oxazolidinone-sulphonamide/amide conjugates that were recently reported by our research group. The antibacterial assays showed that, from the series, oxazolidinones **2** and **3a** exhibited excellent potency (MIC of 1.17 μg/mL) against *B. subtilis* and *P. aeruginosa* strains, along with good antibiofilm activity. Docking studies revealed higher binding affinities of oxazolidinones **2** and **3a** compared to linezolid, which were further validated by molecular dynamics simulations. In addition to this, other computational studies, one-descriptor (log *P*) analysis, ADME-T and drug likeness studies demonstrated the potential of these novel linezolid-based oxazolidinones to be taken forward for further studies.

## 1. Introduction

**Figure 1:**
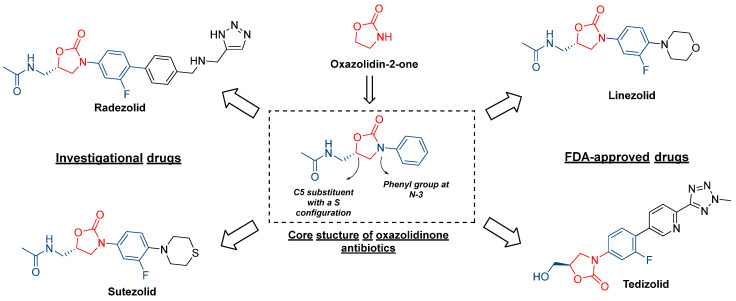
Development of oxazolidinone-based drugs.

Bacterial infections are a growing cause of concern because of the increasing mortality rate. The tiny bacteria are resilient, as they develop resistance to drugs over a period of time [1,2]. Sulfonamides are celebrated as the first antibacterial agents; however, drug-resistant bacterial strains develop within a few years of their use [3]. Over the decades, many different classes of antibacterial agents were discovered and designed to treat bacterial infections, but the emergence of resistance rendered them less potent [4]. Oxazolidinones are one of the new classes of synthetic antibacterial agents compared to other antibacterial drugs [5,6,7]. The core structural feature of this class of antibiotic is a five-membered 2-oxazolidone ring with a C5 substituent in *S* stereo configuration, and a phenyl ring attached to nitrogen at the third position [8]. Mechanistically, the oxazolidinones exert biological effects by targeting the 50S ribosomal units and associated enzymes, such as ribosomal peptidyl-transferases, leading to the disruption of the critical protein synthesis process. Linezolid is the first oxazolidinone antibiotic that was developed and approved by the FDA at the start of the millennium for treating Gram-positive bacterial infections [9]. Moreover, it is also clinically used in treating multidrug-resistant tuberculosis [10], and is a lead molecule in the design and development of new oxazolidinone-based antibiotics (Figure 1). Another oxazolidinone approved as a drug by the FDA is tedizolid, which is used for the treatment of acute infections of the skin by bacteria [11]. Some other oxazolidinone antibiotics are in different stages of clinical trials, such as sutezolid, radezolid, delpazolid, TBI-223, etc., and a few were not pursued further because of different issues that occurred during clinical trials [12,13,14]. In addition to this, several research groups have designed new oxazolidinone derivatives and evaluated their antibacterial potency, with good degrees of success [15,16,17,18].

**Figure 2:**
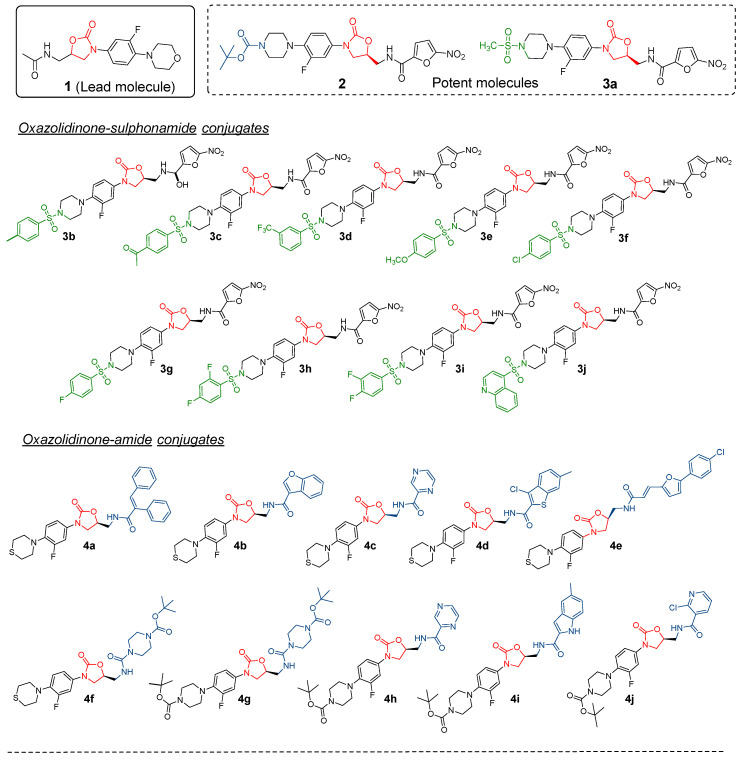
Design of novel linezolid-based oxazolidinones [24].

In view of our research interest in the development of new bioactive compounds [19,20,21,22,23], we recently reported the design and synthesis of novel linezolid-based oxazolidinones as anticandidal and antitubercular agents [24]. In our previous study, the lead molecule (linezolid **1**) was modified by the replacement of thiomorpholine with *N*-sulphonyl piperazine moiety, incorporation of a 2-nitrofuranamide group and the replacement of acetamide with heteryl and biphenylacryl amides. The synthesis of the designed novel linezolid-based oxazolidinones were carried out through a key precursor, an oxazolidinone compound **2,** resulting in a series of oxazolidinone-sulphonamide conjugates (**3a–j**) and oxazolidinone-amide conjugates (**4a–j**) (Figure 2). These novel conjugates were subjected to profiling of their anti-fungal activities, and the oxazolidinones **2** and **3a** showed three- to several-fold better antifungal activity than the standards miconazole and fluconazole. Mechanistic and computational studies further highlighted the potential of these conjugates. In this study, we report the antibacterial potency of these novel linezolid-based oxazolidinones (**3, 4a–j**) and the understanding of their mode of action using computational tools, which included docking and molecular dynamic simulations at the molecular level. Moreover, a quantitative one-descriptor (logP) analysis model was developed to further highlight the potential of these oxazolidinones.

## 2. Results and Discussion

### 2.1. Antibacterial Evaluation

The novel linezolid-based oxazolidinones with sulphonamide/amide conjugates (**3a–j** and **4a–j**) were synthesized employing a multistep synthetic route that resulted in good yields, as recently reported by our research group [24]. The anticandidal and antitubercular activities of these conjugates were reported earlier [24], and in the present study, we explored the antibacterial potency of these oxazolidinones (**2**, **3a–j, 4a–j**) against different bacterial strains, which included both Gram-positive and Gram-negative strains, with neomycin as a positive control (Table 1). The results showed that oxazolidinone-sulphonamides (**3a–j**) and oxazolidinone-amides (**4a–j**) demonstrated enhanced potency against Gram-positive bacteria compared to Gram-negative counterparts (Table 2). They were most active toward *Bacillus subtilis* MTCC 121 and *Staphylococcus aureus* MTCC 2940 strains, with most of the MIC values in the range of 1.17–37.5 µg/mL. In another strain of *Staphylococcus aureus* MTCC 96, the activity was not as pronounced, with MIC values between 4.68 and 125 µg/mL. Intriguingly, no activity was seen against other Gram-positive *Micrococcus luteus* strains. In the case of the Gram-negative bacterial strains, the conjugates were active only against *Pseudomonas aeruginosa* MTCC 2453, with MIC values in the range of 1.17–75 µg/mL. No activity was observed against *Klebsiella planticola* and *Escherichia coli* strains. Among the series of compounds, conjugate **3a** was the most active, displaying the best activity and an MIC value of 1.1 µg/mL against *Bacillus subtilis* and *Pseudomonas aeruginosa* strains. The oxazolidinone with the nitrofuran moiety was potent [25]; hence, we also investigated intermediate **2**, it also exhibited the best MIC value of 1.17 µg/mL against three strains. Both conjugates **2** and **3a** were much more potent than neomycin, the standard used. This encouraged us to further study the potency of conjugates **2** and **3a**, and we evaluated their bactericidal potential against three different bacterial strains. The results showed that the oxazolidinone **2** demonstrated a good minimum bactericidal concentration (MBC) value of 2.34 μg/mL against *Bacillus subtilis* and *Staphylococcus aureus* MLS-16, along with an MBC value of 4.68 μg/mL against *Pseudomonas aeruginosa* MTCC 2453. On the other hand, oxazolidinone **3a** exhibited MBC values of 4.68 and 9.36 μg/mL against the tested strains.

### 2.2. Structure–Activity Relationship

The experimental data were used to understand the structure–activity relationship (SAR), which showed some interesting patterns. In general, both the oxazolidinone-sulphonamides/amides (**3**, **4a–j**) showed identical activity patterns towards the different bacterial strains, with no activity against *Micrococcus luteus*, *Klebsiella planticola* and *Escherichia coli* strains. Nonetheless, the most active compound from the series was an oxazolidinone with sulphonamide conjugation, i.e., **3a**. In oxazolidinone-sulphonamides **3a–j**, the compound with a simple methyl substitution (**3a**) displayed more potency compared to aromatic substitutions, with MIC values between 1.17 to 18.75 µg/mL against four bacterial strains. The sulphonamides with 4-methylphenyl (**3b**) and 4-acetylphenyl (**3c**) groups were the second-best, with MIC values in the range of 2.34–37.5 µg/mL. The halogen substitutions [3-trifluoromethylphenyl (**3d**), 4-chlorophenyl (**3f**), 2,4-difluorophenyl (**3h**), 3,4-difluorophenyl (**3i**), and 4-chlorophenyl (**3g**)] lowered the activity, with MIC values between 9.36 and >75 µg/mL, and the exception was **3f**, which displayed good activity against *Staphylococcus aureus* and *Pseudomonas aeruginosa* strains. The conjugate with the 4-methoxyphenyl substitution exhibited good activity (MIC: 4.36 µg/mL) against *Staphylococcus aureus* MTCC 96. Interestingly, the lone conjugate with the quinoline substitution (**3j**) also exhibited good activity against *Bacillus subtilis* and *Pseudomonas aeruginosa*, with MIC values of 2.34 and 4.68 µg/mL, respectively.

In the case of the oxazolidinone-amide series, it was clearly evident that the conjugates with the thiomorpholine ring exhibited better activity than conjugates with the *N*-Boc piperazine moiety. The conjugates with heterocyclic rings, pyrazine (**4c**), benzothiophene (**4d**) and substituted furan (**4e**) were the most active from the series against *Bacillus substilis* and *Pseudomonas aeruginosa*, as well as *Staphylococcus aureus* MTCC 2940 (MIC: 2.34 and 4.68 µg/mL). The conjugate **4d** was an exception that showed an MIC value of 18.75 µg/mL against the *Staphylococcus aureus* strain. Conjugate **4i** with the indole ring also displayed good activity (MIC: 4.68 µg/mL) against the *Staphylococcus aureus* MTCC 2940 and *Pseudomonas aeruginosa* strains. The diphenyl acryloyl and *N*-Boc piperazine moieties present in conjugates **4a** and **4g** also displayed decent activity against *Bacillus substilis* (MIC: 9.36 µg/mL). The other conjugates incorporating heterocyclic rings, such as benzofuran (**4b**), piperazine (**4f**), pyrazine without the thiomorpholine group (**4h**), were not very active against all of the tested strains.

### 2.3. Antibiofilm Evaluation

The formation of biofilm, an extracellular polymer-based matrix, is a defensive mechanism adapted by a spectrum of microorganisms to protect themselves against antimicrobial agents [26]. Bacterial biofilms have been reported to cause chronic infections and exhibit resistance to antibiotics [27]. The potent oxazolidinones conjugates (**3**, **2a**) were also tested for antibiofilm activity against *Bacillus subtilis*, *Staphylococcus aureus* MLS- 16 and *Pseudomonas aeruginosa*. The results showed that the conjugates exhibited good inhibition of biofilms against the tested strains (Table 3). Oxazolidinone **2** exhibited significantly higher antibiofilm activity than oxazolidinone **3a**, with an IC_50_ value of 0.58 µg/mL against *Bacillus subtilis* and *Pseudomonas aeruginosa*. On the other hand, conjugate **3a** displayed IC_50_ values between 1.24 and 2.34 µg/mL.

### 2.4. Molecular Docking Studies

**Figure 3:**
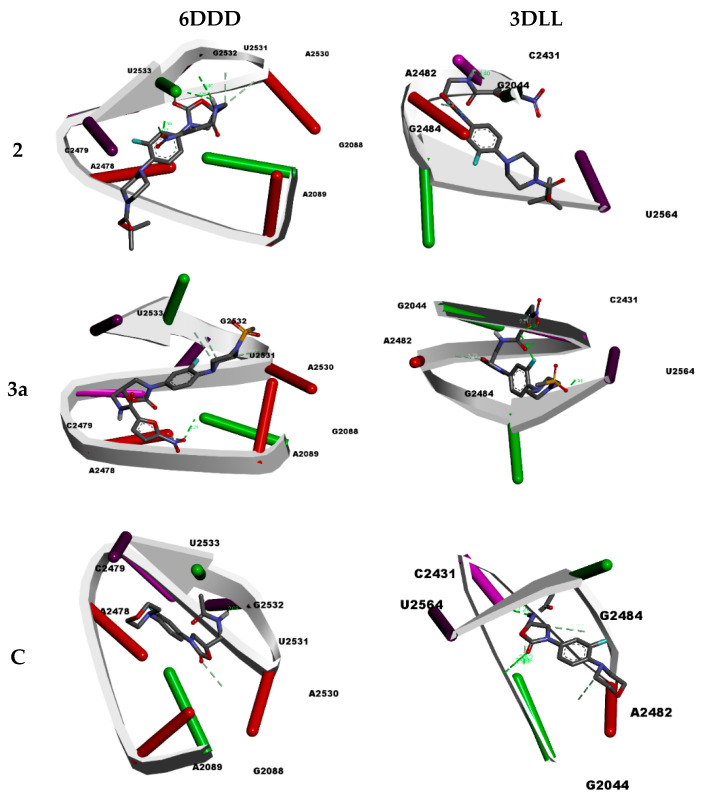
Binding interactions of the potent oxazolidinones (**2** and **3a**) with 50S ribosomal units (6DDD), and the associated ribosomal peptidyl-transferase (3DLL). The control used was linezolid (C).

Oxazolidinone antibiotics are known to display potent antibacterial agents by the inhibition of the crucial protein synthesis required for viable bacteria [5]. The 50S ribosomal units and the associated ribosomal peptidyl-transferase protein belong to the molecular machinery that works toward the synthesis of life-sustaining bacterial proteins [28]. Therefore, the potent antibacterial properties of oxazolidinones **2** and **3a** were subjected to docking studies, in order to understand their molecular interactions with the 50S ribosomal units (6DDD) [29] and the associated ribosomal peptidyl-transferase protein (3DLL) [30]. The positive control used was linezolid, a clinically used oxazolidinone-based drug (Figure 3). The docking studies revealed interesting results; oxazolidinones **2** and **3a** showed enhanced binding affinities towards the molecular targets than the linezolid control.

The studies with the 50S ribosomal unit molecular target (6DDD) revealed that oxazolidinone **2** displayed the best results, with a binding energy score of −6.36 kcal/mol and a 187.94 uM inhibition constant score (Table 4). The binding interaction primarily resulted from the formation of multiple (seven) hydrogen bonds between oxazolidinone **2** and the target receptor. The hydrogen bond lengths were in the range of 1.82 to 3.38 Å between the nucleotide residues, G2532:H3, G2532:H21, G2532:O5’, G2532:O4’, G2532:C4’, A2530:O2’, G2532:OP2 and the different atoms on oxazolidinone **2** (UNK0:O7, UNK0:O7, UNK0:H1, UNK0:H1, UNK0:O1, UNK0:C1, UNK0:C1) [31]. The UNK0 represents the chemical ligand. In case of oxazolidinone **3a**, the binding energy and inhibition constant values of −6.01 kcal/mol and 557.88 uM, respectively, were observed, resulting from six hydrogen bond formations (2.28–3.74 Å). The hydrogen bond formations happened between the nucleotide residues, G2088:H22, A2478:C2, A2478:C2, G2532:O5’, A2530:O2’, G2532:O4’ and different atoms on oxazolidinone **3a** (UNK0:O7, UNK0:O5, UNK0:O7, UNK0:C12, UNK0:C11, UNK0:C9). The control, linezolid, showed a lowered binding affinity, with a −5.41 kcal/mol binding energy value with only two hydrogen bond formations.

On the other hand, the docking studies with ribosomal peptidyl-transferase (3DLL) revealed that oxazolidinone **3a** had better affinity than oxazolidinone **2** and linezolid. It exhibited a −6.61 kcal/mol binding energy score and a 297.30 uM inhibition constant. Six hydrogen bond formations (1.93–3.21 Å) were observed between the nucleoside residues G2044:H22, G2044:H22, U2564:HO2’, G2044:O6, A2482:C8, G2044:H1, and different atoms on oxazolidinone **3a** (UNK0:F, UNK0:O6, UNK0:O3, UNK0:H1, UNK0:O1, UNK0:O). In the case of oxazolidinone **2**, a binding energy score of −6.37 kcal/mol was observed, with a good inhibition constant score (170.07 uM). Only four hydrogen bond formations (1.80–3.15 Å) between the nucleotide residues G2044:H1, G2044:O6, A2482:C1’, A2482:C8 and atoms UNK0:O5, UNK0:H1, UNK0:O1 and UNK0:O1 were revealed. The control, linezolid, also showed a lower binding affinity with the ribosomal peptidyl-transferase (3DLL) target than oxazolidinone **2** and **3a**, with a binding energy score of −5.41 kcal/mol. Interestingly, linezolid showed multiple hydrogen bond formations, with bond lengths in the range of 2.52–3.77 Å. In addition to hydrogen bond formations, few other non-covalent interactions, such as π-sigma, π-π stacking, π-alkyl, π-lone pair and π-π T-shaped, were observed.

### 2.5. Molecular Dynamics Simulations

**Figure 4:**
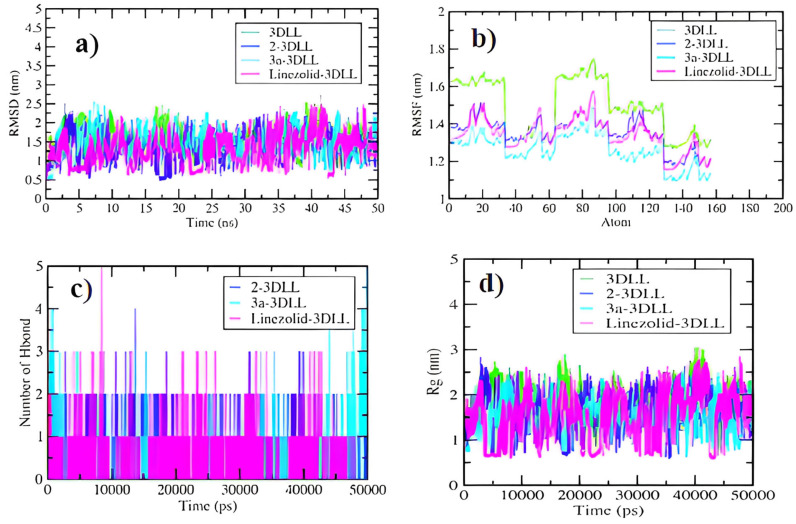
2D plots obtained in the MDS study. (**a**) RMSD plot of 3DLL in water (green), **2**-3DLL (blue), **3a**-3DLL (turquois) and linezolid-3DLL (pink) complexes. (**b**) RMSF plot showing the fluctuations per residue. (**c**) Hydrogen bond plot representing the number of hydrogen bond formations. (**d**) Radius of gyration (Rg) plot representing compactness of the complexes.

The docking results encouraged us to undertake a molecular dynamic simulation study, in order to predict the binding stabilities between ligands (**2**, **3a**, linezolid) and ribosomal peptidyl-transferase (3DLL). This led us to understand the positions of different atoms and molecules during the binding of the ligands with receptors, which are helpful in drug design [32]. A 50-nanosecond MDS experimentation run was conducted and analyzed for RMSD, RMSF, radius of gyration and a hydrogen bond formation plot. The obtained data showed the deviation and the fluctuation of ribosomal peptidyl-transferase (3DLL) in water and its complexes with oxazolidinone (**2**-3DLL, **3a**-3DLL) and linezolid-3DLL during the entire simulation period. The root mean square deviation provides a measure of the average distance between the atoms of the protein–ligand complexes. In the present study, the RMSD values were in the range of 0.5–2.5 nm throughout the period for all complexes. Interestingly, 3DLL complexation with oxazolidinones **2** and **3a** displayed a stable pattern as compared to linezolid-3DLL and 3DLL in water simulations (Figure 4A). In the case of RMSF calculations, the values per residues were in the range of 1.1 to 1.6 nm, and fluctuations were observed at the 10–20, 50, 80–90, 110 and 140–150 regions of the residues. It was also observed that **2**-3DLL, **3a**-3DLL and linezolid-3DLL complexes showed lesser values between 1.1–1.5 nm as compared to 3DLL simulation in water, showing values between 1.4–1.6 nm (Figure 4B). The hydrogen bond plot revealed the presence of 1–5 hydrogen bonds during the 50 ns MDS experiment (Figure 4C). The radius of gyration analysis (RoG) highlighted the compactness and stability of the structure of the molecular targets throughout the simulation period. The observed average value of radius of gyration values were 0.5–3.0 nm. It was observed that 3DLL in water, **2**-3DLL, **3a**-3DLL and linezolid-3DLL complexes exhibited the same stability pattern for the whole simulation period, and the compactness was maintained (Figure 4D). The analysis of the radius of gyration showed that **2**-3DLL, **3a**-3DLL and linezolid-3DLL complexes had approximately similar values during the 50000 ps simulation.

### 2.6. One-Descriptor (log P) Analysis

**Figure 5:**
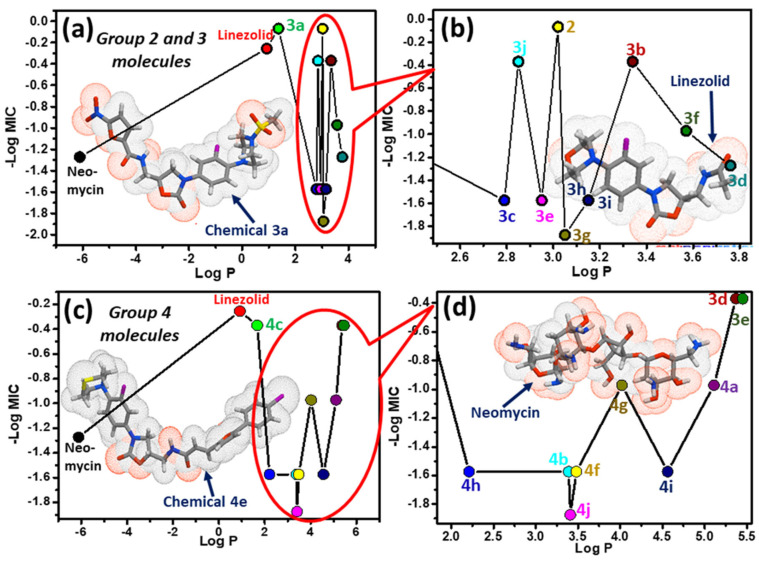
One-descriptor (log *P*) analysis of the potential antibacterial agents—minimum inhibitory concentration (−log MIC) vs. log P. (**a**) For molecules of group 2 and 3 (**b**) Zoomed view of the red circled molecules of panel ‘a’ (**c**) For molecules of group 4 (**d**) Zoomed view of the red circled molecules of panel ‘c’.

In recent decades, computational tools are becoming indispensable to expedite the lengthy process of drug design and development [33]. Three-dimensional quantitative structure-activity relationships provide statistical models that highlight the connection between the physicochemical parameters and the biological potency of chemical substances, especially their lipophilicity and solubility, which are responsible for drug absorption [34,35,36]. The lipophilic nature of the drug controls its absorption in the body, due to the phospholipid-based permeable property of the gastrointestinal epithelial cells. The lipophilicity of a drug also regulates metabolic functions, as it has a tendency to have a higher affinity towards metabolic enzymes [37]. This property also controls its binding with proteins, and its distribution throughout the body. Usually, the more lipophilic the drug is, the stronger is its chance to bind with protein, and the higher the chance of its distribution [38]. Hence, for the present study, initially the one-descriptor lipophilicity or partition coefficient (log *P*) parameter was explored by plotting minimum inhibitory concentration (−log MIC) vs. log *P* of the potential antibacterial agents. Figure 5a,b represent the data set for oxazolidinone-sulphonamide (**3a–j**) and Figure 5c,d represent the data set for oxazolidinone-amide conjugates (**4a–j**). Compounds **2**, **3a**, **3b**, **3j** and **4c**, **4d**, **4e** showed impressive MIC values, which were very close to the MIC value of the standard drug, linezolid. It is interesting to note that molecules **2** and **3a**, containing *tert*-butyl, nitrofuran and piperazine groups, respectively, had better antibacterial activity compared to standard linezolid. In the plot, the lowest antibacterial activities in the 3 and 4 series were found to be **3g** and **4j**, containing the fluorophenylsulfonyl and methyl-1*H*-indole-2-carboxamido moieties.

Using the linear regression model, it was found that for both oxazolidinone conjugates (**3**, **4a–j**), the biological activity was well correlated (correlation factor 0.6) with lipophilicity (log *P*), within a specific range of log *P* (2 < log *P* < 5.4). The outlier molecules whose log *P* values were beyond the specified range (**3a**, **4c**, **4e**), may need other descriptors for QSAR analysis. One of the significant molecular property descriptors, topological polar surface area (TPSA), was also analyzed in the present study. TPSA is a very fast method for PSA calculation, using the 2D structure of the molecule [39]. PSA is the sum of surfaces of polar atoms, nitrogen and oxygen present in a compound, and it gives good correlations with absorptions of drugs through gastrointestinal absorption [40], Caco-2 permeability [41] and blood–brain barrier crossing [42]. In the present study, it was observed that for oxazolidinone-amide conjugates (**4a–j**), the TPSA values were moderately correlated with their antimicrobial properties. After detailed analysis, it was found that for oxazolidinone-sulphonamide conjugates (**3a–j**), one of the MOE-type descriptors (PEOEVSA6), which are basically the fragments of topological polar surface area, showed a moderate correlation with the MIC values.

**Figure 6:**
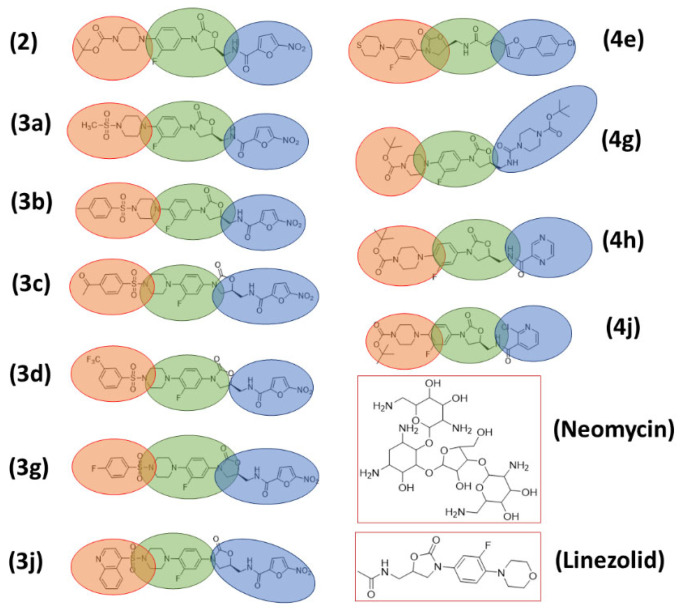
Functional group-based decomposition of oxazolidinone.

**Figure 7:**
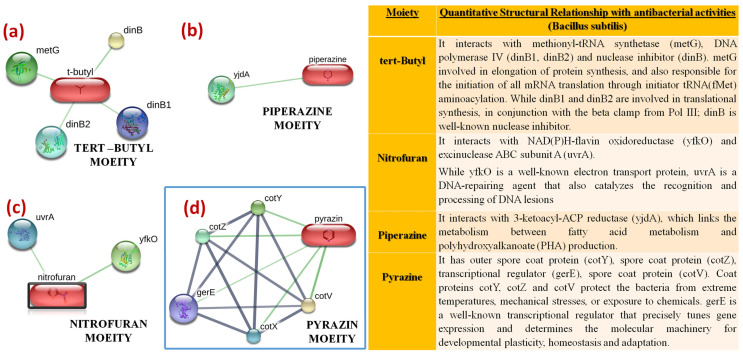
Quantitative biological relationships of various moieties of oxazolidinones (**a**) tert-Butyl moiety (**b**) Piperazine moiety (**c**) Nitrofuran moiety (**d**) Pyrazine moiety.

In drug discovery programs, the identification of drug–target interaction networks is a crucial step; however, this is a very slow and costly process. In silico predictions are an alternative that provide useful and timely information [43]. In addition to docking studies, we studied the chemical–protein interaction networks of the functional groups, using the STITCH database. The functional group-based decomposition of oxazolidinone conjugates (**3**, **4a–j**) was carried out (Figure 6). Moreover, the chemical moieties in the oxazolidinone conjugates, i.e., tert-butyl, nitrofuran, piperazine and pyrazine groups may contribute significantly to enhancing the antibacterial effect and the related antibacterial activities of the chemical moieties, as described in Figure 7.

### 2.7. ADME-T and Drug Likeness Analyses

The major hurdles encountered by the newly synthesized drugs are the pharmacotherapeutic challenges, which could be associated either with toxicity or poor absorption of the drugs [44]. To find out the performance status of the molecules, an ADMET (absorption, distribution, metabolism, excretion and toxicity)-based computational study was conducted (Table 5). It was evident that the majority of oxazolidinone-amide conjugates exhibited high gastrointestinal (GI) tract absorption, which makes them suitable as potential drug candidates. It was also observed from the given table that no drug from the list of compounds showed the ability to penetrate the brain–blood barrier; however, all of them showed a positive p-gp substrate property. Positive p-gp substrate activity indirectly offers drug-like properties. For example, p-glycoprotein (p-gp) plays an important role as a drug transporter in multidrug resistance and pharmacokinetics. Strong affinity of the synthesized molecule may offer a notable strategy for combating multidrug-resistant diseases [45]. The cytochrome P450 (CYP) enzyme has a unique function of expelling foreign bodies such as carcinogens, pesticides and other hazardous chemicals present in the body. In addition, they also play a critical role in the metabolism of drugs, which are foreign bodies. The inhibition of these enzymes helps in understanding the metabolism of drugs [46,47]. The analysis showed that oxazolidinones **2** and **3a–j** showed inhibition of CYP2C19 and CYP3A4 isoforms, and no inhibition of CYP1A2 and CYP2D6 isoforms. In the case of isoform CYP2C19, an interesting observation was that only the active conjugates **2** and **3a** showed inhibition, and the remaining conjugates showed no inhibition. The CYP isoform inhibition profiles of conjugates **4a–j** were substantially different from those of conjugates **3a–j**. They all inhibited the CYP2C19, CYP2D6 and CYP3A4 isoforms, except **4h**, which did not inhibit CYP2D6. A heterogenous pattern was seen in the inhibition profile of other isoforms, namely CYP1A2 and CYP2C9. The toxicity predictions of all of the conjugates were also carried out (see Supplementary Materials Tables S1–S6).

The drug likeness analysis of the conjugates was carried out against the five different filter rules (Lipinski, Ghose, Veber, Egan, Muegge), with interesting results. Conjugates **2**, **3a–j** showed two violations of the Lipinski and Ghose filters, and a single violation of the Egan and Muegge filters. The active conjugates **2** and **3a** also showed a single violation of the Muegge filter. The drug likeness properties of conjugates **4a–j** were better compared to conjugates **3a–j**, with most of the conjugates showing no violations or a single violation against the five drug filters. In particular, conjugates **4b** and **4c** passed all of the five drug filters, similarly to the control. The bioactivity score of 0.17 was predicted for all **3a–j** conjugates, and for a few of the conjugates for the **4a–j** series. Interestingly, most of the conjugates in the **4a–j** series showed bioactive scores of 0.55, similarly to the control.

## 3. Experimental Section

### 3.1. Biological Evaluation

#### 3.1.1. Antibacterial Assay

The antibacterial properties of oxazolidinone **2** and the oxazolidinone-sulphonamides/amides (**3**, **4a–j**) were determined by the well diffusion method (Lindsay method) against Gram-positive strains (*Bacillus subtilis* MTCC 121, *Staphylococcus aureus* MTCC 2940 and MTCC 96, *Micrococcus luteus* MTCC 2470) and Gram-negative strains (*Pseudomonas aeruginosa* MTCC 2453, *Klebsiella planticola* MTCC 530, *Escherichia coli* MTCC 739). A few identical colonies were transferred from the master agar plate into a tube with 5 mL of nutrient agar. This bacterial inoculum was streaked over agar plates, using a sterile cotton swab. To ensure a uniform spreading, the step was repeated several times by a 60-degree rotation of the plates followed by swabbing of the rim of the agar. The plates were allowed to dry at room temperature, and sterilized cork bore was used to make wells with 6 mm diameters. Different concentrations of test compounds in dimethyl sulfoxide (DMSO) were prepared (0.25–125 µg/mL) and introduced into duplicate wells. The incubation of the agar plates was carried out at 37 °C for 24 h and studied for the inhibition of bacterial growth. The inhibition zone diameters (IZD) were measured to the nearest millimeter.

#### 3.1.2. MBC Assay

Bactericidal assays were carried out in 2 mL microfuge tubes against the three most susceptible strains (*Bacillus subtilis* MTCC 121, *Staphylococcus aureus* MLS-16 MTCC 2940 and *Pseudomonas aeruginosa* MTCC 2453). The strains were first cultured overnight in MH (Mueller–Hinton) broth, followed by the preparation of serial dilutions of the test conjugates (**3** and **2a**) in MH broth with different concentrations. To the test conjugates, 100 µL of bacterial suspensions were added to reach a final concentration of 0.5 McFarland, followed by incubation at 37 °C for 24 h. After 24 h of incubation, the minimum bactericidal concentrations (MBCs) were determined by sampling 10 µL of suspension from the tubes onto Mueller–Hinton agar plates, and were incubated for 24 h at 37 °C to observe the growth of the test organisms. The MBC is the lowest concentration of compound required to kill a particular bacterium. All of the experiments were carried out in duplicate, and were represented by their mean values.

#### 3.1.3. Biofilm Inhibition Assay

The test conjugates (**3** and **2a**) were screened against *Bacillus subtilis* MTCC 121, *Staphylococcus aureus* MLS-16 and *Pseudomonas aeruginosa* MTCC 2453 in 96-well polystyrene microtiter plates. The bacterial strains were cultured overnight in tryptone soy broth with 0.5% glucose, and predetermined concentrations of test conjugates ranging from 0 to 250 µg/mL were mixed with an inoculum of bacteria (5 × 10^5^ CFU/mL). Then, 100 µL aliquots were added into each well, and the plates were incubated at 37 °C for 24 h. After incubation, the medium was removed, and the contents were washed with saline to remove the non-adherent bacteria. Staining was carried out by incubating each well with 100 µL of 0.1% crystal violet solution for 30 min, followed by discarding the crystal violet solution from the plates. The plates were washed with distilled H_2_O, and then dried at ambient temperature. The stained biofilm was then dissolved in 95% ethanol (100 µL) and measured for its absorbance at 540 nm, using a TRIAD multimode reader. Dose–response curves were generated to obtain IC_50_ values, and reported as the mean ± S.D. of three experiments.

### 3.2. Computational Studies

#### 3.2.1. Molecular Docking

Molecular docking was executed between conjugates **2** and **3a** and 50S ribosomal units (6DDD), as well as the associated ribosomal peptidyl-transferase protein (3DLL), using AutoDock 4.2 software. The control used in the study was linezolid. The Lamarckian Genetic Algorithm (LGA) and empirical binding free energy function were used by AutoDock as a scoring function for the ligand–receptor interactions [48]. The default AutoDock parameters were used for the execution, but a 60 × 60 × 60 Å grid box was used to cover the maximum area. After docking, the results were examined, and Discovery Studio Visualizer 2019 was used to generate the graphics.

#### 3.2.2. Molecular Dynamics Simulations

MD simulations of the target receptor 3DLL were carried out in water and in complexation with conjugate **2**, **3a** and linezolid, employing the methodology adopted in our previous study [49]. The MDS environment was settled up to execute 50 nanosecond (ns) simulations using the GROMACS tool 2018 version [50]. The pdb2gmx module was used to generate the required 3DLL topology file, followed by selection of the CHARMM27 all-atom force field. Then, chemical compounds **2**, **3a**, and linezolid topology files were generated from the SwissParam server, followed by solvation, stabilization and energy minimization. The equilibrium setup of the system for **2**-3DLL, **3a**-3DLL and linezolid-3DLL complexes was followed by two-step ensembles NVT and NPT. The GORMACS gmx rms package was used for root mean square deviation (RMSD) gmx rmsf for root mean square fluctuation (RMSF), gmx gyrate for the calculation of radius of gyration (Rg) and gmx hbond for the calculation of numbers of hydrogen bonds formed during the interactions. Finally, after successful 50 ns simulation runs, trajectory files and graphical plots were generated using the xmgrace program.

#### 3.2.3. Descriptor (log *P*) Analysis, ADME-T and Drug Likeness Analyses

We computed physicochemical descriptors as well as the predictive ADMET parameters, pharmacokinetic properties and drug-like natures of the newly synthesized compounds. We followed the methodology described in earlier research [51]. The chemical–protein interaction networks of the functional groups were studied using the STITCH database [52]. The STITCH Consortium2016 (http://stitch.embl.de/,accessed on 29 October 2022) web resource (version 5.0) was used to predict chemical–protein (CP) interaction networks of the functional groups for the novel antibiotics. About 430,000 compounds and 960,000 proteins curated from 2031 eukaryotic and prokaryotic genomes can be predicted using the STITCH database [53]. The confidence score, where a larger score indicates a stronger interaction, can be used to predict the relationship for a chemical–protein interaction. For this investigation, a medium confidence score of (0.4) was applied. The active interactions were populated from eight distinct sources, including experiments, text mining, neighbourhood, gene fusion, databases, co-expression, co-occurrence and predictions.

## 4. Conclusions

Oxazolidinones are a relatively new class of synthetic antibiotics, with only two drugs approved by the FDA. The emergence of drug resistance to different antibiotics demands the development of new antibacterial agents. We reported the anticandidal and antitubercular activities of our novel linezolid-based oxazolidinones. The bacterial studies showed that oxazolidinone **2** and **3a** were the most potent, with MIC and MBC values of 1.1–4.68 µg/mL against *B. subtilis*, along with good antibiofilm activity. The SAR showed that in conjugates **3a–j**, a simple methyl substitution (**3a**) was more potent compared to aromatic substitutions, while a thiomorpholine ring exhibited better activity in conjugates **4a–j**. Docking studies revealed the binding energy scores of the potent conjugates (**2**, **3a**) were better than that of linezolid, and the molecular docking simulations demonstrated that the conjugates targeted receptors similarly to linezolid. The one-descriptor (log *P*) analysis was used to analyze the biological activities of the novel oxazolidinone. Many of these descriptors were also used to calculate the ADMET properties of the compounds. The studies showed that the novel linezolid-based oxazolidinones have significant antibacterial properties, with the potential to be taken up further for in vivo studies.

## Figures and Tables

**Table 1:** Antibacterial activities of oxazolidinone-sulfonamide conjugates (**3a–j**) ^1^.

Compound	*B. s* ^2^	*S. m* ^2^	*S. a* ^2^	*M. l* ^2^	*P. a* ^3^	*K. p* ^3^	*E. c* ^3^
**2**	1.17 (2.34) ^4^	1.17 (2.34) ^4^	125	>125	1.17 (4.68) ^4^	>125	>125
**3a**	1.17 (4.68) ^4^	4.68 (9.36) ^4^	18.75	>125	1.17 (4.68) ^4^	>125	>125
**3b**	2.34	18.75	37.5	>125	4.68	>125	>125
**3c**	37.5	4.68	37.5	>125	4.68	>125	>125
**3d**	18.75	75	125	>125	75	>125	>125
**3e**	37.5	>125	4.68	>125	>125	>125	>125
**3f**	9.36	9.36	4.68	>125	4.68	>125	>125
**3g**	125	37.5	125	>125	75	>125	>125
**3h**	37.5	18.75	37.5	>125	18.75	>125	>125
**3i**	37.5	18.75	37.5	>125	18.75	>125	>125
**3j**	2.34	9.36	>125	>125	4.68	>125	>125
Nmyn ^5^	18.75	18.75	18.75	18.75	18.75	18.75	18.75

^1^ Antibacterial activity is reported as minimum inhibition concentration (MIC, µg/mL), and the values are the means of triplicates; ^2^ Gram-positive bacterial strain; ^3^ Gram-negative bacterial strain; ^4^ minimum bactericidal concentration (MBC) values; ^5^ neomycin used as control; *B. s* (*Bacillus subtilis* MTCC 121); *S. m* (*Staphylococcus aureus* MLS-16 MTCC 2940); *S. a* (*Staphylococcus aureus* MTCC 96); *M. l* (*Micrococcus luteus* MTCC 2470); *P. a* (*Pseudomonas aeruginosa* MTCC 2453); *K. p* (*Klebsiella planticola* MTCC 530); *E. c* (*Escherichia coli* MTCC 739).

**Table 2:** Antibacterial activities of oxazolidinone-amide conjugates (**4a–j**) ^1^.

Compound	*B. s* ^2^	*S. m* ^2^	*S. a* ^2^	*M. l* ^2^	*P. a* ^3^	*K. p* ^3^	*E. c* ^3^
**4a**	9.36	75	18.75	>125	3.37	>125	>125
**4b**	37.5	18.75	37.5	>125	18.75	>125	>125
**4c**	2.34	4.68	18.75	>125	4.68	>125	>125
**4d**	2.34	18.75	37.5	>125	4.68	>125	>125
**4e**	2.34	4.68	37.5	>125	4.68	>125	>125
**4f**	37.5	18.75	37.5	>125	18.75	>125	>125
**4g**	9.36	75	>125	>125	75	>125	>125
**4h**	37.5	18.75	37.5	>125	18.75	>125	>125
**4i**	37.5	4.68	37.5	>125	4.68	>125	>125
**4j**	>125	>125	>125	>125	>125	>125	>125
Nmyn ^4^	18.75	18.75	18.75	18.75	18.75	18.75	18.75

^1^ Antibacterial activity is reported as minimum inhibition concentration (MIC, µg/mL) and the values are the means of triplicates; ^2^ Gram-positive bacterial strain; ^3^ Gram-negative bacterial strain; ^4^ neomycin used as control; *B. s* (*Bacillus subtilis* MTCC 121); *S. m* (*Staphylococcus aureus* MLS-16 MTCC 2940); *S. a* (*Staphylococcus aureus* MTCC 96); *M. l* (*Micrococcus luteus* MTCC 2470); *P. a* (*Pseudomonas aeruginosa* MTCC 2453); *K. p* (*Klebsiella planticola* MTCC 530); *E. c* (*Escherichia coli* MTCC 739).

**Table 3:** Antibiofilm activities of the oxazolidinone derivatives, **2** and **3a**.

S. No.	Compound	IC_50_ Values (in μg/mL)		
		*Bacillus subtilis* MTCC 121	*Staphylococcus aureus* MLS16 MTCC 2940	*Pseudomonas aeruginosa* MTCC 2453
1	**2**	0.58 ± 0.18	1.21 ± 0.32	0.58 ± 0.08
2	**3a**	1.24 ± 0.09	2.32 ± 0.28	2.34 ± 0.26
3	Erythromycin	0.22 ± 0.14	0.25 ± 0.12	0.19 ± 0.22

**Table 4:** Docking analysis of active conjugates (**2** and **3a**) with ribosomal-based molecular targets.

S. No.	Compound	Molecular Target	Final Intermolecular Energy (kcal/mol)	Inhibition Constant	Hydrogen Bonds *	H-Bond Length (Å)	Other Interactions
1.	**2**	6DDD	−6.36	187.94 uM	1:G2532:H3–:UNK0:O7 1:G2532:H21–:UNK0:O7 :UNK0:H1–1:G2532:O5’ :UNK0:H1–1:G2532:O4’ 1:G2532:C4’–:UNK0:O1 :UNK0:C1–1:A2530:O2’ :UNK0:C1–1:G2532:OP2	1.82 2.43 2.79 2.35 3.38 3.69 2.96	1:G2088 = π-sigma π-π stacking
2.	**3a**	6DDD	−6.01	557.88 uM	1:G2088:H22–:UNK0:O7 1:A2478:C2–:UNK0:O5 1:A2478:C2–:UNK0:O7 :UNK0:C12–1:G2532:O5’ :UNK0:C11–1:A2530:O2’ :UNK0:C9–1:G2532:O4’	2.28 2.96 3.34 3.10 3.36 3.74	1:A2530 = π-alkyl
3.	**Linezolid (C)**	6DDD	−5.41	182.26 uM	:UNK0:H–1:G2532:O5’ 1:A2530:C8–:UNK0:O	2.08 2.91	1:U2533 = π-alkyl 1:A2578 = π-lone pair
4.	**2**	3DLL	−6.37	170.07 uM	X:G2044:H1–:UNK0:O5 :UNK0:H1–X:G2044:O6 X:A2482:C1’–:UNK0:O1 X:A2482:C8–:UNK0:O1	1.80 2.40 2.98 3.15	C:X2431 π-π T-shaped
5.	**3a**	3DLL	−6.61	297.30 uM	X:G2044:H22–:UNK0:F X:G2044:H22–:UNK0:O6 X:U2564:HO2’–UNK0:O3 :UNK0:H1–X:G2044:O6 X:A2482:C8–:UNK0:O1 X:G2044:H1–:UNK0	2.83 1.97 1.93 1.95 3.21 2.70	C:X2431 π-π T-shaped
6.	**Linezolid (C)**	3DLL	−4.92	377.31 uM	X:G2044:H22–:UNK0:O X:G2044:H1–:UNK0:O :UNK0:H–X:C2431:N3 :UNK0:C–X:G2484:OP2 :UNK0:C–X:G2044:O6 :UNK0:C–X:A2482:N7	2.89 2.52 2.28 3.77 3.45 3.47	A:X2482 = π-sigma, π-alkyl G:X2044 = π-π T-shaped

* Where X and 1 represent the nucleotide chain; UNK0 represents the chemical ligand.

**Table 5:** Selected ADME parameters of oxazolidinones **2**, **3a–j**, **4a–j**.

Compounds	GI Absorption	BBB Permeant	Pgp Substrate	CYP1A2 Inhibitor	CYP2C19 Inhibitor	CYP2C9 Inhibitor	CYP2D6 Inhibitor	CYP3A4 Inhibitor
**2**	Low	No	Yes	No	Yes	Yes	No	Yes
**3a**	Low	No	Yes	No	Yes	Yes	No	Yes
**3b**	Low	No	Yes	No	No	Yes	No	Yes
**3c**	Low	No	Yes	No	No	Yes	No	Yes
**3d**	Low	No	Yes	No	No	Yes	No	Yes
**3e**	Low	No	Yes	No	No	Yes	No	Yes
**3f**	Low	No	Yes	No	No	Yes	No	Yes
**3g**	Low	No	Yes	No	No	Yes	No	Yes
**3h**	Low	No	Yes	No	No	Yes	No	Yes
**3i**	Low	No	Yes	No	No	Yes	No	Yes
**3j**	Low	No	Yes	No	No	Yes	No	Yes
**4a**	High	No	Yes	No	Yes	Yes	Yes	Yes
**4b**	High	No	Yes	Yes	Yes	Yes	Yes	Yes
**4c**	High	No	Yes	No	Yes	No	Yes	Yes
**4d**	Low	No	Yes	Yes	Yes	Yes	Yes	Yes
**4e**	High	No	Yes	No	Yes	Yes	Yes	Yes
**4f**	High	No	Yes	No	Yes	No	Yes	Yes
**4g**	High	No	Yes	No	Yes	Yes	Yes	Yes
**4h**	High	No	Yes	No	Yes	Yes	No	Yes
**4i**	High	No	Yes	No	Yes	Yes	Yes	Yes
**4j**	High	No	Yes	No	Yes	Yes	Yes	Yes

## Data Availability

Data is contained within the article and supplementary material.

## Supplementary Materials

The following supporting information can be downloaded at: https://www.mdpi.com/article/10.3390/ph16040516/s1, Table S1: Physicochemical properties of compounds **2**, **3a-j**, **4a-j** and linezolid (control); Table S2: Lipophilicity and water solubility of compounds **2**, **3a-j**, **4a-j** and linezolid (control); Table S3: Some other water solubility parameters of compounds **2**, **3a-j**, **4a-j** and linezolid (control); Table S4: Some other water solubility parameters of compounds **2**, **3a-j**, **4a-j** and linezolid (control); Table S5: Drug likeness and medicinal chemistry of compounds **2**, **3a-j**, **4a-j** and linezolid (control); Table S6: Toxicity of compounds **2**, **3a-j**, **4a-j** and linezolid (control).
