# Supplementary material for: Antibacterial Properties and Computational Insights of Potent Novel Linezolid-Based Oxazolidinones
Source: Pharmaceuticals (Basel). 2023 Mar 30;16(4):516. doi: 10.3390/ph16040516 (PMC10143092; doi:10.3390/ph16040516)
Supplement: Supplementary file 1 [file pharmaceuticals-16-00516-s001.zip › pharmaceuticals-2288100-supplementary.pdf]

Supporting Information For:

Antibacterial Properties and Computational Insights of Potent Novel Linezolid-Based Oxazolidinones

M. Shaheer Malik [1,*,†], Shaikh Faazil [2,3,†], Meshari A. Alsharif [1], Qazi Mohammad Sajid Jamal [4], Jabir H. Al-Fahemi [1], Amrita Banerjee [5,6], Arpita Chattopadhyay [7], Samir Kumar Pal [8], Ahmed Kamal [3,9,*] and Saleh A. Ahmed [1,10,*]

1 Department of Chemistry, Faculty of Applied Sciences, Umm Al-Qura University, Makkah 21955, Saudi Arabia; maasharif@uqu.edu.sa (M.A.A.)
2 Department of Chemistry, Poona College of Arts, Science and Commerce, Pune 411001, India
3 Department of Medicinal Chemistry and Pharmacology, CSIR—Indian Institute of Chemical Technology, Hyderabad 500007, India
4 Department of Health Informatics, College of Public Health and Health Informatics, Qassim University, Al Bukayriyah 52741, Saudi Arabia; m.quazi@qu.edu.sa
5 Department of Physics, Jadavpur University, 188, Raja S.C. Mallick Rd., Kolkata 700032, India; amritabanerjee30@gmail.com
6 Technical Research Centre, S. N. Bose National Centre for Basic Sciences, Block JD, Sector III, Salt Lake, Kolkata 700106, India
7 Department of Basic Science and Humanities, Techno International New Town, Block—DG 1/1, Action Area 1, New Town, Rajarhat, Kolkata 700156, India; arpita.chattopadhyay@gmail.com
8 Department of Chemical and Biological Sciences, S. N. Bose National Centre for Basic Sciences, Block JD, Sector 3, Salt Lake, Kolkata 700106, India; skpal@bose.res.in
9 Department of Pharmacy, Birla Institute of Technology and Science-Pilani, Hyderabad 500078, India
10 Chemistry Department, Faculty of Science, Assiut University, Assiut 71516, Egypt
* Correspondence: msmalik@uqu.edu.sa (M.S.M.); ahmedkamal@iict.res.in (A.K.); saahmed@uqu.edu.sa (S.A.A.)
† These authors contributed equally to this work.

Keywords: Oxazolidinone, Linezolid, antibacterial, molecular modeling, molecular dynamics, ADME properties

Contents:

- **Table S1**: Physicochemical properties of compounds **2**, **3a-j**, **4a-j** and linezolid (control)
- **Table S2**: Lipophilicity and water solubility of compounds **2**, **3a-j**, **4a-j** and linezolid (control)
- **Table S3**: Some other water solubility parameters of compounds **2**, **3a-j**, **4a-j** and linezolid (control)
- **Table S4**: Pharmacokinetics parameters of compounds **2**, **3a-j**, **4a-j** and linezolid (control)
- **Table S5**: Drug likeness and medicinal chemistry of compounds **2**, **3a-j**, **4a-j** and linezolid (control)
- **Table S6**: Toxicity of compounds **2**, **3a-j**, **4a-j** and linezolid (control)

Table S1. Physicochemical properties of compounds **2**, **3a-j**, **4a-j** and linezolid (control).

Molecule	#Heavy atoms	#Aromatic heavy atoms	Fraction Csp3	#Rotatable bonds	#H-bond acceptors	#H-bond donors	MR	TPSA
2	38	11	0.46	10	9	1	143.52	150.38
3a	35	11	0.4	8	10	1	131.55	166.6
3b	41	17	0.31	9	10	1	155.36	166.6
3c	43	17	0.3	10	11	1	160.58	183.67
3d	44	17	0.31	10	13	1	155.39	166.6
3e	42	17	0.31	10	11	1	156.88	175.83
3f	41	17	0.28	9	10	1	155.4	166.6
3g	41	17	0.28	9	11	1	150.35	166.6
3h	42	17	0.28	9	12	1	150.31	166.6
3i	42	17	0.28	9	12	1	150.31	166.6
3j	44	21	0.25	9	11	1	165.69	179.49
4a	37	18	0.24	8	4	1	152.46	87.18
4b	32	15	0.3	6	5	1	127.24	100.32
4c	29	12	0.37	6	6	1	113.06	112.96
4d	34	15	0.33	6	4	1	142.83	115.42
4e	37	17	0.26	8	5	1	149.89	100.32
4f	36	6	0.62	9	6	1	149.63	119.96
4g	43	6	0.66	12	8	1	174.6	124.2
4h	36	12	0.46	9	8	1	138.02	117.2
4i	40	15	0.41	9	6	2	159.26	107.21
4j	37	12	0.44	9	7	1	145.24	104.31
Linezolid	24	6	0.5	5	5	1	91.06	71.11

Table S2. Lipophilicity and water solubility of compounds **2**, **3a-j**, **4a-j** and linezolid (control).

Molecule	iLOGP	XLOGP3	WLOGP	MLOGP	Silicos-IT Log P	Consensus Log P	ESOL Log S	ESOL Solubility (mg/ml)	ESOL Solubility (mol/l)
**2**	3.53	2.94	2.42	1.67	-0.48	2.02	-4.55	1.49E-02	2.79E-05
**3a**	2.45	1.34	1.52	0.34	-1.99	0.73	-3.56	1.41E-01	2.75E-04
**3b**	3.4	3.27	3.26	1.39	-0.46	2.17	-5.26	3.26E-03	5.55E-06
**3c**	2.74	2.58	3.15	0.78	-0.5	1.75	-4.91	7.49E-03	1.22E-05
**3d**	3.08	3.78	5.12	1.69	0.12	2.76	-5.82	9.60E-04	1.50E-06
**3e**	3.2	2.87	2.96	0.92	-0.91	1.81	-5.03	5.64E-03	9.34E-06
**3f**	3.34	3.53	3.61	1.66	-0.34	2.36	-5.55	1.73E-03	2.84E-06
**3g**	2.77	3	3.51	1.56	-0.56	2.06	-5.11	4.59E-03	7.76E-06
**3h**	2.83	3.1	4.07	1.93	-0.13	2.36	-5.28	3.22E-03	5.28E-06
**3i**	3.04	3.1	4.07	1.93	-0.13	2.4	-5.28	3.22E-03	5.28E-06
**3j**	2.81	3.17	3.5	1.14	-0.57	2.01	-5.47	2.12E-03	3.40E-06
**4a**	4.18	5.21	4.61	4.04	4.71	4.55	-6.16	3.55E-04	6.86E-07
**4b**	3.22	3.55	3.54	2.71	3.23	3.25	-4.85	6.41E-03	1.41E-05
**4c**	2.53	1.34	1.58	0.93	1.73	1.62	-3.18	2.74E-01	6.57E-04
**4d**	4.2	5.49	4.97	4.2	5.64	4.9	-6.45	1.83E-04	3.52E-07
**4e**	4.32	4.96	5	3.35	5.02	4.53	-6.14	3.95E-04	7.29E-07
**4f**	3.46	2.16	1.86	2.39	1.17	2.21	-3.98	5.53E-02	1.06E-04
**4g**	5.33	2.39	1.99	2.26	0.66	2.53	-4.42	2.32E-02	3.82E-05
**4h**	3.27	1.57	1.71	0.85	1.13	1.71	-3.59	1.30E-01	2.60E-04
**4i**	4.02	4.21	3.71	2.73	3.33	3.6	-5.6	1.40E-03	2.54E-06
**4j**	3.25	3.27	2.96	2.29	2.35	2.82	-4.86	7.43E-03	1.39E-05
Linezolid	2.58	0.69	0.78	0.99	1.25	1.26	-2.22	2.03E+00	6.01E-03

Table S3. Some other water solubility parameters of compounds **2**, **3a-j**, **4a-j** and linezolid (control).

Molecule	ESOL Class	Ali Log S	Ali Solubility (mg/ml)	Ali Solubility (mol/l)	Ali Class	Silicos-IT LogSw	Silicos-IT Solubility (mg/ml)	Silicos-IT Solubility (mol/l)	Silicos-IT class
**2**	Moderately soluble	-5.76	9.27E-04	1.74E-06	Moderately soluble	-4.22	3.21E-02	6.01E-05	Moderately soluble
**3a**	Soluble	-4.44	1.86E-02	3.63E-05	Moderately soluble	-3.66	1.12E-01	2.18E-04	Soluble
**3b**	Moderately soluble	-6.44	2.12E-04	3.61E-07	Poorly soluble	-6.08	4.85E-04	8.26E-07	Poorly soluble
**3c**	Moderately soluble	-6.09	5.06E-04	8.21E-07	Poorly soluble	-5.99	6.24E-04	1.01E-06	Moderately soluble
**3d**	Moderately soluble	-6.97	6.84E-05	1.07E-07	Poorly soluble	-6.51	1.99E-04	3.10E-07	Poorly soluble
**3e**	Moderately soluble	-6.22	3.62E-04	6.00E-07	Poorly soluble	-5.8	9.52E-04	1.58E-06	Moderately soluble
**3f**	Moderately soluble	-6.71	1.18E-04	1.94E-07	Poorly soluble	-6.29	3.15E-04	5.19E-07	Poorly soluble
**3g**	Moderately soluble	-6.16	4.07E-04	6.87E-07	Poorly soluble	-5.97	6.34E-04	1.07E-06	Moderately soluble
**3h**	Moderately soluble	-6.27	3.30E-04	5.41E-07	Poorly soluble	-6.22	3.63E-04	5.96E-07	Poorly soluble
**3i**	Moderately soluble	-6.27	3.30E-04	5.41E-07	Poorly soluble	-6.22	3.63E-04	5.96E-07	Poorly soluble
**3j**	Moderately soluble	-6.61	1.53E-04	2.45E-07	Poorly soluble	-6.95	7.02E-05	1.12E-07	Poorly soluble
**4a**	Poorly soluble	-6.79	8.43E-05	1.63E-07	Poorly soluble	-8.13	3.85E-06	7.45E-09	Poorly soluble
**4b**	Moderately soluble	-5.34	2.07E-03	4.55E-06	Moderately soluble	-6.48	1.51E-04	3.32E-07	Poorly soluble
**4c**	Soluble	-3.31	2.03E-01	4.85E-04	Soluble	-4.88	5.48E-03	1.31E-05	Moderately soluble
**4d**	Poorly soluble	-7.67	1.11E-05	2.13E-08	Poorly soluble	-7.47	1.77E-05	3.41E-08	Poorly soluble
**4e**	Poorly soluble	-6.8	8.49E-05	1.57E-07	Poorly soluble	-7.93	6.38E-06	1.18E-08	Poorly soluble
**4f**	Soluble	-4.31	2.55E-02	4.88E-05	Moderately soluble	-3.68	1.10E-01	2.09E-04	Soluble
**4g**	Moderately soluble	-4.64	1.39E-02	2.29E-05	Moderately soluble	-3.7	1.21E-01	2.00E-04	Soluble
**4h**	Soluble	-3.64	1.14E-01	2.28E-04	Soluble	-4.92	5.99E-03	1.20E-05	Moderately soluble
**4i**	Moderately soluble	-6.17	3.72E-04	6.74E-07	Poorly soluble	-6.88	7.29E-05	1.32E-07	Poorly soluble
**4j**	Moderately soluble	-5.13	3.91E-03	7.33E-06	Moderately soluble	-5.87	7.19E-04	1.35E-06	Moderately soluble
Linezolid	Soluble	-1.76	5.86E+00	1.74E-02	Very soluble	-3.19	2.18E-01	6.47E-04	Soluble

Table S4. Pharmacokinetics parameters of compounds **2**, **3a-j**, **4a-j** and linezolid (control).

Molecule	GI absorption	BBB permeant	Pgp substrate	CYP1A2 inhibitor	CYP2C19 inhibitor	CYP2C9 inhibitor	CYP2D6 inhibitor	CYP3A4 inhibitor	log Kp (cm/s)
**2**	Low	No	Yes	No	Yes	Yes	No	Yes	-7.47
**3a**	Low	No	Yes	No	Yes	Yes	No	Yes	-8.47
**3b**	Low	No	Yes	No	No	Yes	No	Yes	-7.56
**3c**	Low	No	Yes	No	No	Yes	No	Yes	-8.22
**3d**	Low	No	Yes	No	No	Yes	No	Yes	-7.53
**3e**	Low	No	Yes	No	No	Yes	No	Yes	-7.94
**3f**	Low	No	Yes	No	No	Yes	No	Yes	-7.5
**3g**	Low	No	Yes	No	No	Yes	No	Yes	-7.78
**3h**	Low	No	Yes	No	No	Yes	No	Yes	-7.82
**3i**	Low	No	Yes	No	No	Yes	No	Yes	-7.82
**3j**	Low	No	Yes	No	No	Yes	No	Yes	-7.86
**4a**	High	No	Yes	No	Yes	Yes	Yes	Yes	-5.76
**4b**	High	No	Yes	Yes	Yes	Yes	Yes	Yes	-6.56
**4c**	High	No	Yes	No	Yes	No	Yes	Yes	-7.9
**4d**	Low	No	Yes	Yes	Yes	Yes	Yes	Yes	-5.57
**4e**	High	No	Yes	No	Yes	Yes	Yes	Yes	-6.08
**4f**	High	No	Yes	No	Yes	No	Yes	Yes	-7.96
**4g**	High	No	Yes	No	Yes	Yes	Yes	Yes	-8.3
**4h**	High	No	Yes	No	Yes	Yes	No	Yes	-8.24
**4i**	High	No	Yes	No	Yes	Yes	Yes	Yes	-6.68
**4j**	High	No	Yes	No	Yes	Yes	Yes	Yes	-7.24
Linezolid	High	No	Yes	No	No	No	No	No	-7.87

Table S5. Drug likeness and medicinal chemistry of compounds **2**, **3a-j**, **4a-j** and linezolid (control).

Molecule	Lipinski #violations	Ghose #violations	Veber #violations	Egan #violations	Muegge #violations	Bioavailability Score	PAINS #alerts	Brenk #alerts	Leadlikeness #violations	Synthetic accesibility
**2**	2	2	1	1	1	0.17	1	3	2	4.57
**3a**	2	2	1	1	1	0.17	1	2	2	4.22
**3b**	2	2	1	1	1	0.17	1	2	2	4.63
**3c**	2	2	1	1	3	0.17	1	2	2	4.69
**3d**	2	2	1	1	3	0.17	1	2	3	4.63
**3e**	2	2	1	1	3	0.17	1	2	2	4.63
**3f**	2	2	1	1	2	0.17	1	2	3	4.51
**3g**	2	2	1	1	2	0.17	1	2	2	4.53
**3h**	2	2	1	1	3	0.17	1	2	2	4.56
**3i**	2	2	1	1	3	0.17	1	2	2	4.54

**3j**	2	2	1	1	3	0.17	1	2	2	4.72
**4a**	1	2	0	0	1	0.55	1	2	3	4.31
**4b**	0	0	0	0	0	0.55	1	0	2	3.99
**4c**	0	0	0	0	0	0.55	1	0	1	3.59
**4d**	2	2	0	0	1	0.17	1	0	2	4.1
**4e**	1	2	0	0	0	0.55	1	1	3	4.52
**4f**	1	2	0	0	0	0.55	1	1	2	4.58
**4g**	2	3	1	0	1	0.17	1	1	2	5.19
**4h**	2	2	0	0	0	0.17	1	1	2	4.22
**4i**	1	3	0	0	0	0.55	1	1	3	4.46
**4j**	1	2	0	0	0	0.55	1	2	2	4.19
Linezolid	0	0	0	0	0	0.55	1	0	0	3.32

Table S6. Toxicity of compounds **2**, **3a-j**, **4a-j** and linezolid (control).

**Molecule**	**hERG Blockers**	**Human Hepatotoxicity**	**Drug induced liver injury**	**Ames Toxicity**	**Rat Oral Acute Toxicity**	**FDA Maximum Daily Dose**	**Skin Sensitization**	**Carcinogencity**	**Eye Corrosion**	**Eye Irritation**	**Respiratory Toxicity**
**2**	0.43	0.993	0.982	0.995	0.493	0.249	0.457	0.975	0.003	0.008	0.029
**3a**	0.719	0.993	0.985	0.998	0.371	0.44	0.331	0.968	0.003	0.007	0.187
**3b**	0.817	0.992	0.99	0.998	0.282	0.208	0.18	0.963	0.003	0.007	0.146
**3c**	0.85	0.993	0.992	0.998	0.287	0.216	0.153	0.966	0.003	0.007	0.094
**3d**	0.757	0.994	0.988	0.993	0.363	0.88	0.181	0.949	0.003	0.006	0.341
**3e**	0.855	0.994	0.99	0.998	0.264	0.189	0.157	0.961	0.003	0.007	0.155
**3f**	0.844	0.991	0.99	0.998	0.36	0.175	0.176	0.962	0.003	0.007	0.145
**3g**	0.806	0.996	0.99	0.998	0.26	0.233	0.148	0.965	0.003	0.007	0.205
**3h**	0.64	0.995	0.988	0.997	0.145	0.44	0.166	0.968	0.003	0.007	0.263
**3i**	0.69	0.998	0.989	0.998	0.154	0.394	0.152	0.969	0.003	0.006	0.318
**3j**	0.741	0.992	0.991	0.998	0.321	0.236	0.172	0.956	0.003	0.007	0.15
**4a**	0.409	0.98	0.986	0.619	0.05	0.962	0.281	0.517	0.003	0.009	0.327
**4b**	0.299	0.982	0.969	0.938	0.676	0.758	0.411	0.911	0.003	0.01	0.197
**4c**	0.568	0.984	0.985	0.819	0.055	0.8	0.468	0.575	0.003	0.01	0.072
**4d**	0.138	0.99	0.974	0.93	0.053	0.907	0.814	0.942	0.003	0.011	0.27
**4e**	0.458	0.972	0.979	0.943	0.202	0.527	0.664	0.901	0.003	0.011	0.347
**4f**	0.135	0.992	0.959	0.66	0.219	0.307	0.304	0.937	0.003	0.007	0.016
**4g**	0.084	0.997	0.939	0.348	0.287	0.177	0.234	0.956	0.003	0.005	0.004
**4h**	0.476	0.993	0.983	0.249	0.077	0.365	0.586	0.759	0.003	0.007	0.035
**4i**	0.505	0.993	0.946	0.692	0.529	0.841	0.194	0.894	0.003	0.008	0.06
**4j**	0.427	0.995	0.97	0.754	0.327	0.503	0.209	0.916	0.003	0.007	0.039
Linezolid	0.029	0.971	0.918	0.676	0.042	0.373	0.577	0.725	0.003	0.011	0.046
